# Lactate supplementation modulates molecular and functional responses during chronic neuromuscular electrical stimulation in male rats

**DOI:** 10.14814/phy2.70790

**Published:** 2026-03-04

**Authors:** Toshinori Yoshihara, Yasushi Koriyama, Sakura Ogawa, Hisashi Naito

**Affiliations:** ^1^ Graduate School of Health and Sports Science Juntendo University Chiba Japan; ^2^ Institute of Health and Sports Science & Medicine Juntendo University Chiba Japan

**Keywords:** lactate supplementation, mitochondrial biogenesis, myonuclear, neuromuscular electrical stimulation, protein synthesis

## Abstract

Lactate is increasingly recognized as a signaling molecule that modulates muscle plasticity. We examined the effects of oral L‐sodium lactate supplementation on skeletal muscle adaptation to chronic neuromuscular electrical stimulation (NMES) in rats. Male Wistar rats received oral lactate or water before either a single NMES session (acute) or repeated sessions over 2 weeks (chronic). We assessed muscle weight, strength, myonuclear‐associated protein expression (PCM1), protein synthesis (puromycin incorporation), signaling responses (mechanistic target of rapamycin pathway), and mitochondrial‐related protein expression (PGC‐1α, OXPHOS, and citrate synthase). The oxidative soleus and glycolytic plantaris muscles were analyzed. Lactate supplementation was associated with greater increases in muscle mass and torque during chronic NMES, particularly in the soleus. PCM1 abundance and myofibrillar puromycin incorporation were higher in the lactate‐supplemented group, although there were no significant changes in c‐Myc or rpS6. PGC‐1α expression was elevated in the plantaris muscle, indicating muscle‐type‐specific mitochondrial modulation. However, the expression levels of the lactate transporters (MCT1 and MCT4) and GPR81 remained largely unchanged in response to oral lactate. Collectively, these findings suggest that oral lactate is associated with distinct molecular signatures and functional outcomes during chronic NMES in a muscle type‐dependent manner, warranting further studies using morphological and muscle‐specific functional assessments.

## 1 INTRODUCTION

Skeletal muscle is characterized by considerable plasticity in response to mechanical and metabolic stimuli. For example, resistance training represents a potent anabolic stimulus that promotes muscle hypertrophy and functional enhancement via satellite cell activation, myonuclear accretion, and enhanced protein synthesis (Egerman & Glass, 2014; Snijders et al., 2015). In this context, neuromuscular electrical stimulation (NMES) has been widely employed as an alternative to voluntary resistance training, particularly in clinical and rehabilitation settings, and can induce fiber recruitment and mechanical loading sufficient to stimulate muscle adaptation (Nussbaum et al., 2017). However, its anabolic effects are often limited, particularly when low‐frequency stimulation or short training durations are employed. Consequently, strategies that can contribute to enhancing the efficacy of NMES‐based resistance‐type stimulation are of considerable interest.

Lactate, long considered a mere byproduct of anaerobic metabolism, is now recognized as an important signaling molecule that can have a notable influence on skeletal muscle remodeling. Recent in vitro and in vivo studies have revealed that lactate plays an important role in the activation of key molecular pathways, such as the extracellular signal‐regulated kinase (ERK) and the mechanistic target of rapamycin (mTOR) pathways (Lawson et al., 2022), which are central to mitochondrial biogenesis, angiogenesis, and anabolic growth responses. For example, treatment of cultured myotubes with lactate has been found to promote increases in protein synthesis and induces hypertrophy via ERK signaling (Ohno et al., 2018), and chronic oral intake of lactate in rodents has been shown to be associated with increases in muscle mass and fiber cross‐sectional area, presumably via satellite cell activation and myonuclear accretion (Kyun, Yoo, Park, et al., 2020; Ohno et al., 2019).

However, despite this growing body of evidence, most previous investigations in this field have tended to focus on endurance training models (Takahashi et al., 2019) or on supraphysiological overload paradigms, such as synergist ablation (Shirai et al., 2022), thereby limiting their translational relevance. Moreover, Shirai et al. (2022), who examined the effects of lactate intake following a single bout of NMES‐induced contraction, have reported no enhancement of protein synthesis or related signaling transducers in the gastrocnemius muscle of mice, which thus tends to indicate that the anabolic activity of lactate may be dependent on training context or duration or muscle phenotype. At present, it thus remains to be determined whether lactate supplementation can potentiate skeletal muscle adaptation under physiologically relevant resistance‐type loading, such as that provided by NMES training. Similarly, the fiber‐type‐specific sensitivity of skeletal muscle to lactate signaling under such conditions has yet to be sufficiently assessed. Oxidative and glycolytic muscle fibers differ markedly in their metabolic properties, signaling capacity, and baseline expression of transporters and receptors. For example, compared with glycolytic (fast‐twitch) fibers, oxidative (slow‐twitch) fibers have been established to contain a greater abundance of mitochondria and have higher oxidative enzyme activity (Jingting et al., 2017). In addition, metabolite‐dependent signaling pathways such as mTORC1 and PGC‐1α have been identified as regulators of fiber‐type composition (Bourdeau Julien et al., 2018), and different types of fiber have been found to differ in terms of redox regulation and lactate transporter/receptor expression, which potentially modulate their responses to lactate supplementation (Vasileiadou et al., 2023). Thus, it is plausible that the effects of lactate on skeletal muscle adaptation may differ according to the type of fiber.

Given the aforementioned findings, in the present study, we sought to investigate the effects of oral lactate supplementation on skeletal muscle adaptation to NMES‐induced resistance‐type contraction in male rats. To assess both the early molecular responses and long‐term structural remodeling, we employed a combination acute and chronic stimulation protocols, with a specific focus on myonuclear‐associated protein expression, myofibrillar protein synthesis, mTOR signaling, and markers of mitochondrial‐related signaling in the oxidative soleus and glycolytic plantaris muscles. Furthermore, to evaluate potential shifts in muscle fiber phenotype, we also examined the composition myosin heavy chain (MHC) isoforms. We hypothesized that by promoting anabolic and metabolic remodeling, lactate intake would enhance the adaptation of skeletal muscle to NMES in a fiber‐type‐dependent manner.

## 2 MATERIALS AND METHODS

### 2.1 Animals and ethical approval

As experimental animals, we used Male Wistar rats (10 weeks old) in this study. All animals were housed in a temperature‐controlled environment (24°C ± 1°C) with a 50%–60% humidity under a 12‐h light: dark cycle, and were provided ad libitum access to food (Rodent Lab Diet EQ 5L37, 4.5% fat, 25.0% protein, and 49.3% carbohydrates; Japan SLC, Inc., Hamamatsu, Japan) and water. After 1 week of acclimatization, the rats were randomly assigned to one of the following four groups: (1) water + acute stimulation (WA, *n* = 6), (2) lactate + acute stimulation (LA, *n* = 6), (3) water + chronic stimulation (WC, *n* = 6), and (4) lactate + chronic stimulation (LC, *n* = 7).

All experimental procedures were approved by the Ethics Committee for Animal Experiments at the Sakura Campus, Juntendo University (approval numbers 2024–26 and 2025–28), and were conducted in accordance with the principles for the care and use of laboratory animals stipulated by the Physiological Society of Japan.

### 2.2 Lactate administration

The procedure used to administer lactate was adapted from that previously described with modifications to optimize absorption and timing relative to stimulation (Kyun, Yoo, Hashimoto, et al., 2020; Kyun, Yoo, Park, et al., 2020). L‐sodium lactate (approximately 50% aqueous solution; Cat. No. 195‐05965, FUJIFILM Wako, Japan) was orally administered at a dose of 2 g/kg body weight using a flexible gavage needle (Fuchigami Instruments, Japan) under light isoflurane anesthesia after fasting for 6 h. For rats in both the acute and chronic groups, administration was performed 15–20 min prior to the electrical stimulation session, whereas those in the WA and WC groups received an equal volume of distilled water, which served as the vehicle for lactate administration.

The timing of lactate administration was determined based on preliminary measurements of blood lactate kinetics following the ingestion of oral lactate (2 g/kg). Blood lactate concentrations were assessed at 0, 10, 20, 30, and 60 min post‐administration (*n* = 7). We accordingly detected significant increases in the levels of lactate by 10 min, with concentrations reaching near‐plateau levels by approximately 20 min (Figure S1). On the basis of these observations, to ensure peak systemic availability during muscle contractions, lactate was administered 15–20 min prior to the electrical stimulation sessions.

### 2.3 Electrical stimulation

**FIGURE 1:**
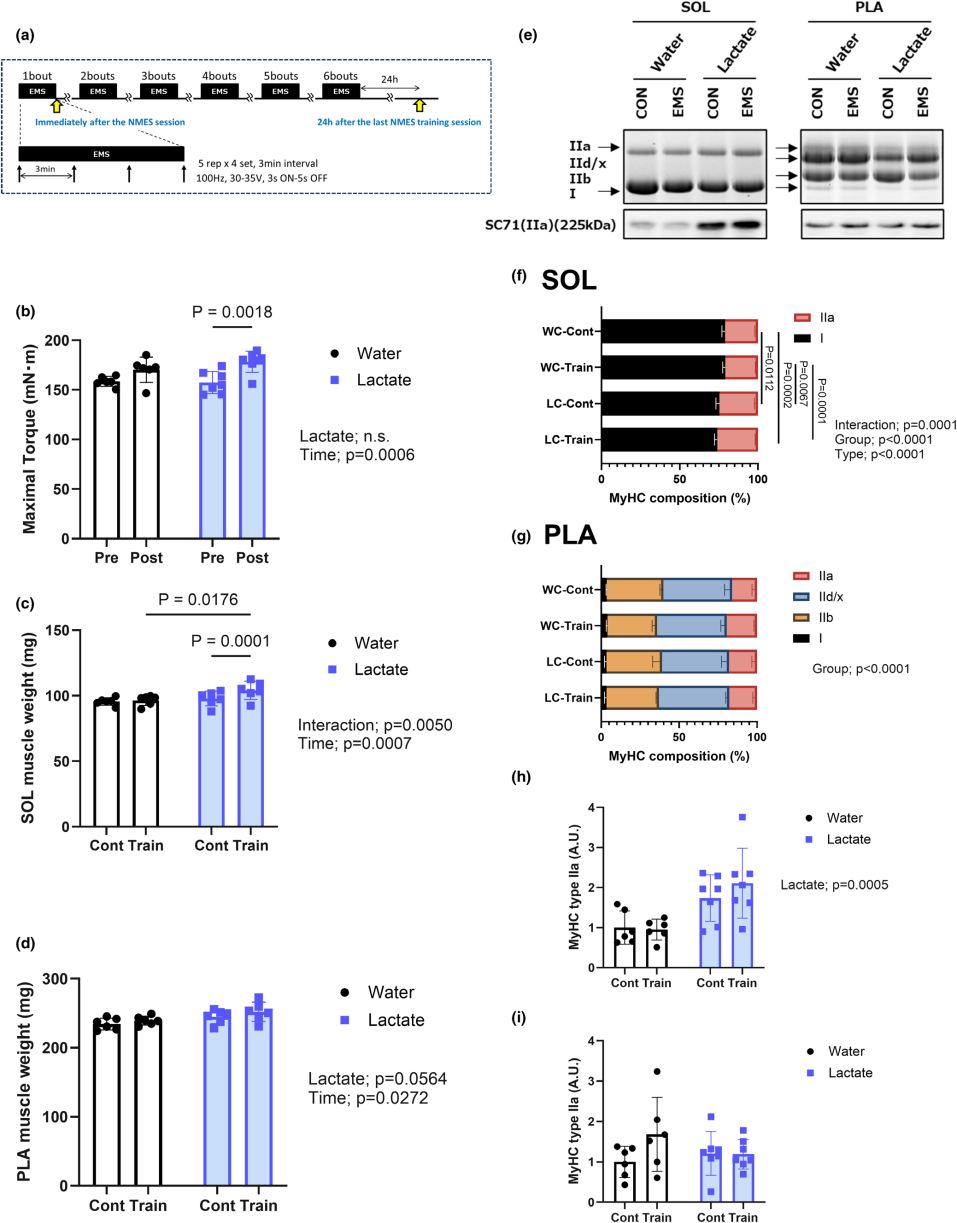
Effects of chronic lactate intake and electrical stimulation on muscle torque, muscle mass, and fiber type composition. (a) Schematic representation of the experimental design. (b) Maximal isometric plantar flexion torque was measured before (Pre) and after (Post) the 2‐week training period in the chronic groups. (c, d) Wet weights of the soleus and plantaris muscles. (e) Representative gel image of myosin heavy chain (MyHC) isoforms in the soleus and plantaris muscles. (f) Relative distribution of MyHC isoforms (types I, IIa, IIx, and IIb) in the soleus muscle. (g) Composition of MyHC isoforms in the plantaris muscle. (h, i) Western blot analysis and quantification of MyHC‐IIa protein in the soleus muscle (h) and plantaris muscle (i). Values are presented as the means ± standard deviation; *n* = 6 or 7 per group. The data were analyzed using two‐way ANOVA followed by Tukey's post hoc test. Additional details of the loading scheme, gel sectioning, membrane assembly, and uncropped immunoblots are provided in Figure S4.

Electrical stimulation was performed under isoflurane anesthesia following a modified version of the protocol described by Tsutaki et al. (2013) (Figure 1a). The rats were placed supine on the custom‐built platform of a small‐animal torque measurement device (T.K.K.5813; Takei Scientific Instruments, Japan), and two self‐adhesive surface electrodes (8 mm × 25 mm) were attached over the triceps surae of one hind limb: one proximal electrode centered over the medial gastrocnemius belly and one distal electrode positioned above the Achilles tendon. To standardize the experimental setup and ensure consistent positioning on the torque measurement platform, neuromuscular electrical stimulation was applied exclusively to the left hind limb in all animals. The contralateral (right) limb served as an unstimulated internal control. Randomization of the stimulation side was not performed to minimize variability from device alignment and limb fixation.

#### 2.3.1 Acute stimulation protocol (WA, LA)

A single session of stimulation was performed, consisting of four sets of tetanic contractions (voltage: 45–65 V; pulse duration: 4 ms; frequency: 100 Hz, 3 s on/5 s off), with 3‐min inter‐set intervals (total 11 min).

#### 2.3.2 Chronic stimulation protocol (WC, LC)

The same stimulation protocol was administered on alternate days for a total of six sessions over 2 weeks (three sessions per week). Torque output was continuously recorded throughout the stimulation using proprietary image analysis software (for T.K.K.5813: ver.1.2.1).

### 2.4 Muscle force assessment

For rats in the chronic stimulation groups (WC and LC), maximal isometric plantar flexion torque was assessed prior to the first and final stimulation sessions. Under anesthesia, the triceps surae were stimulated at 100 Hz with an increasing voltage until maximal torque was reached. Two trials were conducted with a 3‐min inter‐trial interval, and the highest value recorded was considered the maximal isometric torque.

### 2.5 Blood lactate measurements

In both acute and chronic groups, blood samples (~0.3 μL) were collected from the tail vein of rats under brief anesthesia, and lactate levels were measured using a portable lactate analyzer (Lactate Pro 2; Arkray) at baseline, and at 5, 10, 30, and 60 min after stimulation.

On Days 4–5, sampling was limited to Rest, Pre, and 10 min because of constraints related to the repeated sampling burden and anesthesia duration. “Rest” denotes the pre‐ingestion baseline, whereas “Pre” denotes the time point immediately before NMES (approximately 15–20 min after lactate/vehicle administration).

### 2.6 Muscle endurance capacity assessment

To assess the acute effects of lactate ingestion on skeletal muscle endurance, a muscle fatigue resistance test was conducted using a separate cohort of rats (*n* = 6 or 7 per group), independent of those used for the acute and chronic electrical muscular stimulation (EMS) experiments. Following a single oral administration of lactate (2 g/kg, Lactate group) or water (Water group), rats underwent the test after a 20‐min absorption period. Under isoflurane anesthesia, the triceps surae muscles were electrically stimulated at submaximal intensity (45–65 V, 100 Hz, 4 ms) for a total of 25 contractions (3 s on/5 s off). Torque output was continuously recorded throughout the stimulation using a force transducer, and total work was calculated as the area under the torque‐time curve (AUC) using GraphPad Prism (ver. 10.6.1; GraphPad Software Inc., USA). The value thus obtained was used as an index of skeletal muscle endurance capacity.

### 2.7 Blood lactate threshold test

To assess systemic oxidative capacity, a lactate threshold (LT) test was performed in a separate cohort of rats (*n* = 5 or 6 per group), distinct from those used in the acute and chronic EMS experiments. Having orally administered lactate (2 g/kg) or water, rats were placed on a motorized treadmill. The test protocol commenced with an initial running speed of 14 m/min for 3 min, followed by a 2‐min rest period. Thereafter, the running speed was increased by 3 m/min for 3 min, interspersed with 2‐min rest intervals. At the end of each 3‐min running stage, approximately 0.3 μL of blood was collected from the tail vein, and blood lactate concentrations were immediately measured using a portable analyzer (Lactate Pro 2; Arkray). The lactate threshold was defined as the running speed at which blood lactate levels showed a sustained upward trend among stages. In addition, the running speed at which blood lactate concentration reached 4 mmol/L was designated the onset of blood lactate accumulation (OBLA).

### 2.8 Tissue collection and muscle weights

Animals were anesthetized with isoflurane and subsequently euthanized by isoflurane overdose 24 h after the final stimulation (chronic groups) or immediately after a single session (acute groups). The soleus and plantaris muscles were carefully excised from both hindlimbs, weighed, frozen in liquid nitrogen, and stored at −80°C until further analysis.

### 2.9 In vivo protein synthesis measurements

To quantify the rates of protein synthesis in the chronic EMS groups, puromycin (0.04 μmol/g body weight; Cat. No. 160‐23151, FUJIFILM Wako) was injected intraperitoneally 15 min prior to euthanasia, as described previously (Yoshihara et al., 2024). For both soluble and insoluble fractions of the soleus and plantaris muscles, incorporation of puromycin into nascent peptides was assessed based on western blotting using an anti‐puromycin antibody.

### 2.10 Biochemical analyses

#### 2.10.1 Muscle sample preparation and subcellular fractionation

Using a bead mill homogenizer (Microsmash MS‐100; Tomy Seiko, Tokyo, Japan), frozen soleus and plantaris muscle tissue samples (approximately 20 mg) were homogenized in ice‐cold homogenization buffer (20 mM HEPES pH 7.4, 4 mM EGTA, 0.1 mM ethylenediaminetetraacetic acid (EDTA), 10 mM MgCl_2_, and 1% Triton X‐100) containing a protease inhibitor cocktail (cOmplete™ EDTA‐free; Cat. No. 11873580001, Roche Diagnostics, Mannheim, Germany) and phosphatase inhibitor cocktail (PhosSTOP™; Cat. No. 4906845001, Roche). The homogenate was then centrifuged for 15 min at 12,000 × *g* and 4°C, and the resulting supernatant was collected as the soluble fraction. To isolate the myofibrillar protein fraction, the insoluble pellet obtained from cytoplasmic extraction was washed multiple times with 10 volumes of ice‐cold homogenization buffer to remove the residual soluble components. After each wash, samples were centrifuged for 5 min at 12,000 × *g* and 4°C. The final pellet was resuspended in 10 volumes of lysis buffer containing 20 mM HEPES (pH 7.4), 150 mM NaCl, 1% (w/v) lithium dodecyl sulfate (LDS), and 1% (v/v) Nonidet P‐40. The suspension was then centrifuged for 5 min at 17,000 × *g* and 4°C, and the resulting supernatant was collected as the insoluble fraction. Protein concentrations were measured using a bicinchoninic acid protein assay kit (Cat. No. 23225, Thermo Fisher Scientific, Waltham, MA, USA).

#### 2.10.2 Western blot analysis

Equal amounts of protein (10–20 μg) were resolved on 4%–15% TGX Stain‐Free™ pre‐cast polyacrylamide gels (Cat. No. 4568081, Bio‐Rad Laboratories, Hercules, CA, USA) and transferred to PVDF membranes (Cat. No. 1620177, Bio‐Rad), which were subsequently blocked for 20–60 min at 25°C–26°C with blocking buffer (EveryBlot; Cat. No. 12010020, Bio‐Rad) or PVDF Blocking Reagent for Can Get Signal® (Cat. No. NYPBR01, Toyobo, Osaka, Japan).

Thereafter, the membranes were incubated overnight at 4°C with primary antibodies diluted in EveryBlot (Bio‐Rad) or Can Get Signal® immunoreaction enhancer (Cat. No. NKB‐101, Toyobo). As primary antibodies, we used the following: mTOR (1:2000; Cell Signaling, #2983), p‐mTOR (1:2000; Cell Signaling, #2971), 4EBP1 (1:2000; Cell Signaling, #9452), p‐4EBP1 (1:2000; Cell Signaling, #2855), p70S6K (1:2000; Cell Signaling, #9202), p‐p70S6K (1:2000; Cell Signaling, #9234), ERK (1:2000; Cell Signaling, #4695), p‐ERK (1:2000; Cell Signaling, #4370), Puromycin (1:2000; Sigma, MABE343), c‐Myc (1:2000; Cell Signaling, #9402), rpS6 (1:2000; Cell Signaling, #2217), PCM1 (1:2000; Proteintech, 19856‐1‐AP), PGC1α (1:2000; Calbiochem, STR04), OXPHOS (1:2000; Abcam, ab110413), CS (1:2000; Cell Signaling, #14309), MCT1 (1:2000; Proteintech, 20139‐1‐AP), MCT4 (1:2000; Proteintech, 22787‐1‐AP), GPR81 (1:2000; Cosmo Bio, AF24022642‐001), and MyHC Type IIa protein (1:2000: SC‐71; Developmental Studied Hybridoma Bank, University of Iowa, Iowa City, IA, USA). After washing, the membranes were incubated with HRP‐conjugated anti‐rabbit or anti‐mouse secondary antibodies (1:10000; Cell Signaling, #7074 or #7076) for 1 h at 25°C–26°C. Signals were visualized using ECL Prime (Cat. No. RPN2232, GE Healthcare) and imaged using a ChemiDoc™ Touch Imaging System (Bio‐Rad). Band intensities were quantified using Image Lab software (version 5.2.1; Bio‐Rad). Protein load homogeneity was confirmed using Revert 700 Total Protein Stain (Cat. No. 926‐11021, LI‐COR Biosciences, Lincoln, NE, USA). To ensure consistent and interpretable comparisons among experimental groups, levels of protein expression and phosphorylation ratios were normalized to the mean values obtained for the Water control, which were set to 1.0. Schematic illustrations and uncropped immunoblot images are provided in Figures [Link], [Link].

#### 2.10.3 Myosin heavy chain composition

Changes in skeletal muscle fiber type were assessed based on myosin heavy chain (MyHC) composition using a modified version of a previously described method (Ogura et al., 2011). Gels were stained with Coomassie InstantBlue Protein Stain (Abcam, ab119211), scanned using a ChemiDoc Touch Imaging System (Bio‐Rad), and analyzed with Image Lab v.5.2.1 software (Bio‐Rad) to assess the relative percentages of MyHC isoforms.

### 2.11 Statistical analysis

Data are presented as the means ± standard deviation. Two‐way ANOVA (factors: lactate × electrical stimulation or group × fiber type) followed by Tukey's post hoc test was used to compare group differences. Within‐animal comparisons (stimulated vs. non‐stimulated limbs) or within‐group comparisons (Water vs. Lactate groups) were analyzed using paired or non‐paired *t*‐tests. Statistical significance was defined as a *p* value < 0.05. All statistical analyses were performed using GraphPad Prism version 10.6.1 (GraphPad Software, USA).

#### 2.11.1 Sample size and power

The smallest effect size of interest (SESOI) for changes in muscle weight (soleus and plantaris) was predefined as ±6%–8% of the respective control means (M = 100 and 240 mg, respectively). Using historical within‐group SDs of 3 and 9 mg, respectively, with a two‐sided α‐value of 0.05, power of 0.80, and equal allocation, an a priori calculation in GraphPad Prism indicated the required per‐group sample sizes of *n* = 3 or 4 (soleus) and 4–7 (plantaris) across the 6%–8% SESOI range. On the basis of the a priori power calculations, we prospectively set the sample size to *n* = 6 or 7 animals per group, depending on the experiment, thereby providing a conservative margin for potential variance inflation and attrition. In line with the 3Rs, this range was regarded as the minimum number of animals compatible with our predefined SESOI and statistical rigor.

## 3 RESULTS

### 3.1 Muscle force output and muscle weight

Chronic electrical stimulation was found to promote a significant increase in maximal isometric torque in the stimulated limb (main effect of time; *p* = 0.0006) (Figure 1b). Although lactate intake alone did not produce a statistically significant main effect, post hoc analysis revealed a significant increase in post‐training torque in the lactate + chronic stimulation (LC) compared with the corresponding pre‐training value. These findings thus indicate that when combined with electrical muscular stimulation, an intake of lactate may have an additive effect on torque enhancement.

Consistent with these functional changes, we found that the soleus muscle was characterized by a significant Lactate × Time interaction (*p* = 0.0050) and a robust main effect of time (*p* = 0.0007), indicating a greater increase in wet muscle mass among rats in the lactate‐supplemented training group (Figure 1c). Moreover, for the plantaris muscle, although the interaction did not reach a level of statistical significance, both a time effect (*p* < 0.05) and a near‐significant main effect of lactate (*p* = 0.0564) were observed (Figure 1d), implying a potential cumulative benefit of lactate intake on a training‐induced gain in muscle mass.

Electrophoretic separation of MyHC isoforms revealed significant differences among groups with respect to the composition of fiber types in the soleus muscle (Figure 1e). Two‐way ANOVA revealed a significant interaction between treatment group (WC‐cont, WC‐train, LC‐cont, and LC‐train) and fiber type (I, IIa, IIx, and IIb) (interaction: *p* = 0.0001), as well as significant main effects of both group and fiber type (both *p* < 0.0001) (Figure 1f). Post hoc comparisons for the soleus muscle indicated that compared with rats in the WC‐cont and WC‐train groups, those in both the LC‐cont and LC‐train groups were characterized by a substantially greater proportion of type IIa MyHC, thereby indicating a shift toward a more oxidative glycolytic phenotype in response to lactate intake, regardless of training. Further evidence for this shift was obtained based on our Western blot analysis of MyHC‐IIa protein expression in the soleus muscle, which revealed a significant main effect of lactate (*p* = 0.0005) (Figure 1h), thereby confirming an increase in IIa protein abundance among rats in the lactate‐administered groups.

In contrast, the composition of MyHC isoforms in the plantaris muscle showed a significant main effect of group (*p* < 0.0001) (Figure 1g), although on the basis of post hoc comparisons, we failed to detect any significant differences among groups. This would thus tend to indicate that the plantaris muscle, being a predominantly fast‐twitch type, is not characterized by a substantial phenotypic remodeling in response to either training or lactate intake.

### 3.2 Blood lactate responses to oral lactate intake during training sessions

**FIGURE 2:**
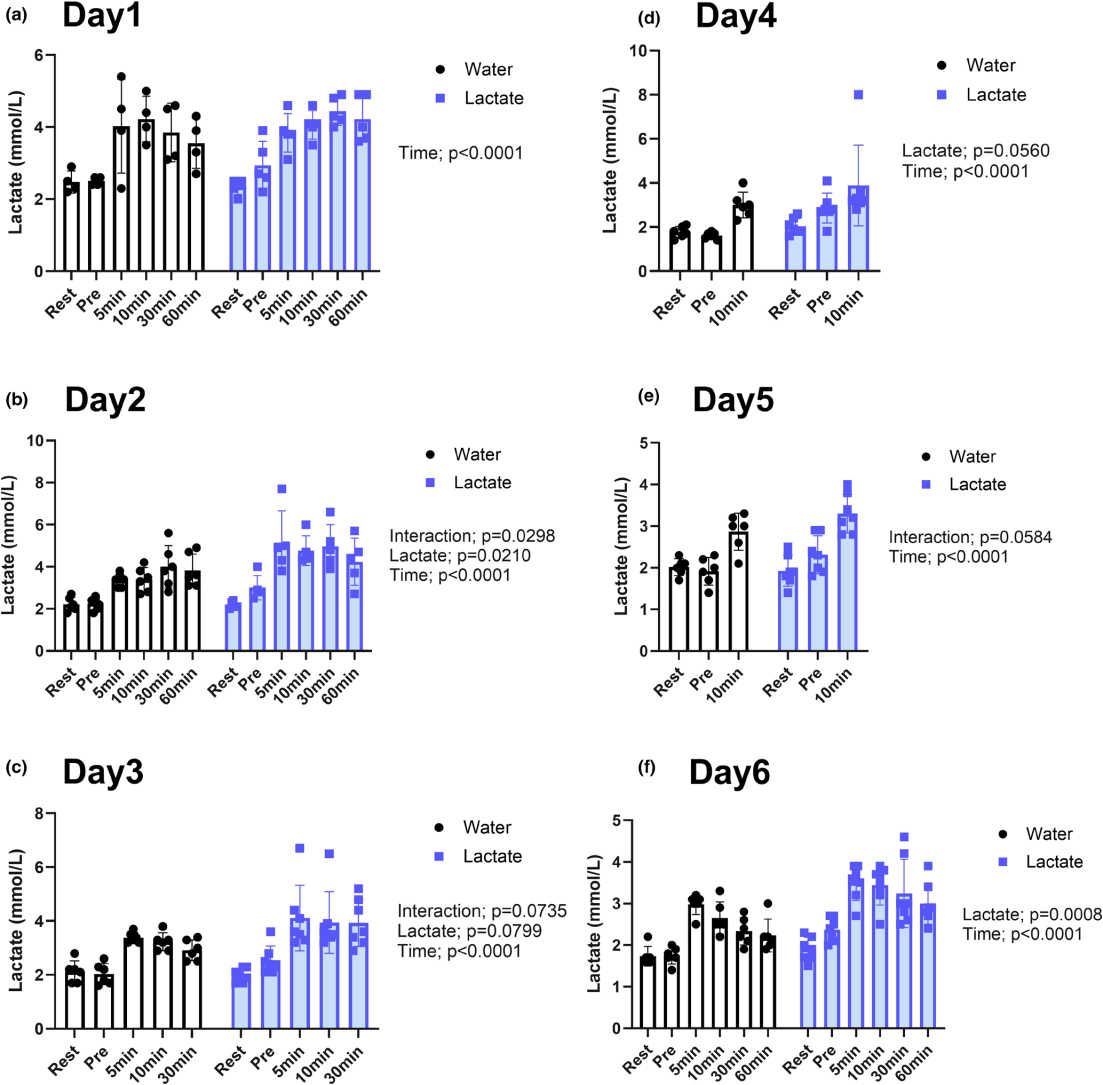
Blood lactate concentrations during acute and chronic stimulation sessions. Time courses of blood lactate levels in response to oral water (WC) or lactate (LC) intake measured before and after electrical stimulation on (a) Day 1, (b) Day 2, (c) Day 3, (d) Day 4, (e) Day 5, and (f) Day 6. Values are presented as the means ± standard deviation; *n* = 6 or 7 per group. The data were analyzed using a two‐way ANOVA.

To evaluate the systemic effects of oral lactate intake, we monitored blood lactate concentrations throughout the training sessions (Figure 2). On Day 1, whereas no significant changes in blood lactate levels were observed in the water‐treated group (WC) over the 20‐min post‐ingestion period (Figure 2a), rats in the lactate + chronic stimulation group (LC) were found to be characterized by a significant increase in blood lactate concentrations subsequent to oral intake (*p* < 0.05), confirming the efficacy of the adopted dosing protocol. On Day 2, we detected a significant interaction between the ingestion of lactate and time (*p* = 0.0298), along with a main effect of lactate (*p* = 0.0210), indicating that lactate further augmented the stimulation‐induced increase in blood lactate levels (Figure 2b). On Day 3, similar trends were observed, with a near‐significant interaction (*p* = 0.0735) and a trend toward a main effect of lactate (*p* = 0.0799) (Figure 2c). On Days 4 and 5, lactate intake showed main effects approaching significance (*p* = 0.0560 and *p* = 0.0584, respectively), and by Day 6, a robust main effect of lactate was evident (*p* = 0.0008) (Figure 2d–f). Among all sessions (Days 1–6), time‐dependent increases in blood lactate levels were consistently observed (main effect of time; all *p* < 0.05).

Owing to differences in the daily durations of sampling (up to 60 min on Days 1, 2, and 6; up to 30 min on Day 3; and up to 10 min on Days 4 and 5), temporal comparisons were limited to available time points within each session. Nevertheless, the consistent elevation in levels of blood lactate in the LC group rats among different sessions provided evidence for the sustained bioavailability of orally administered lactate throughout the intervention period.

### 3.3 Peak torque during training sessions and fatigue resistance

**FIGURE 3:**
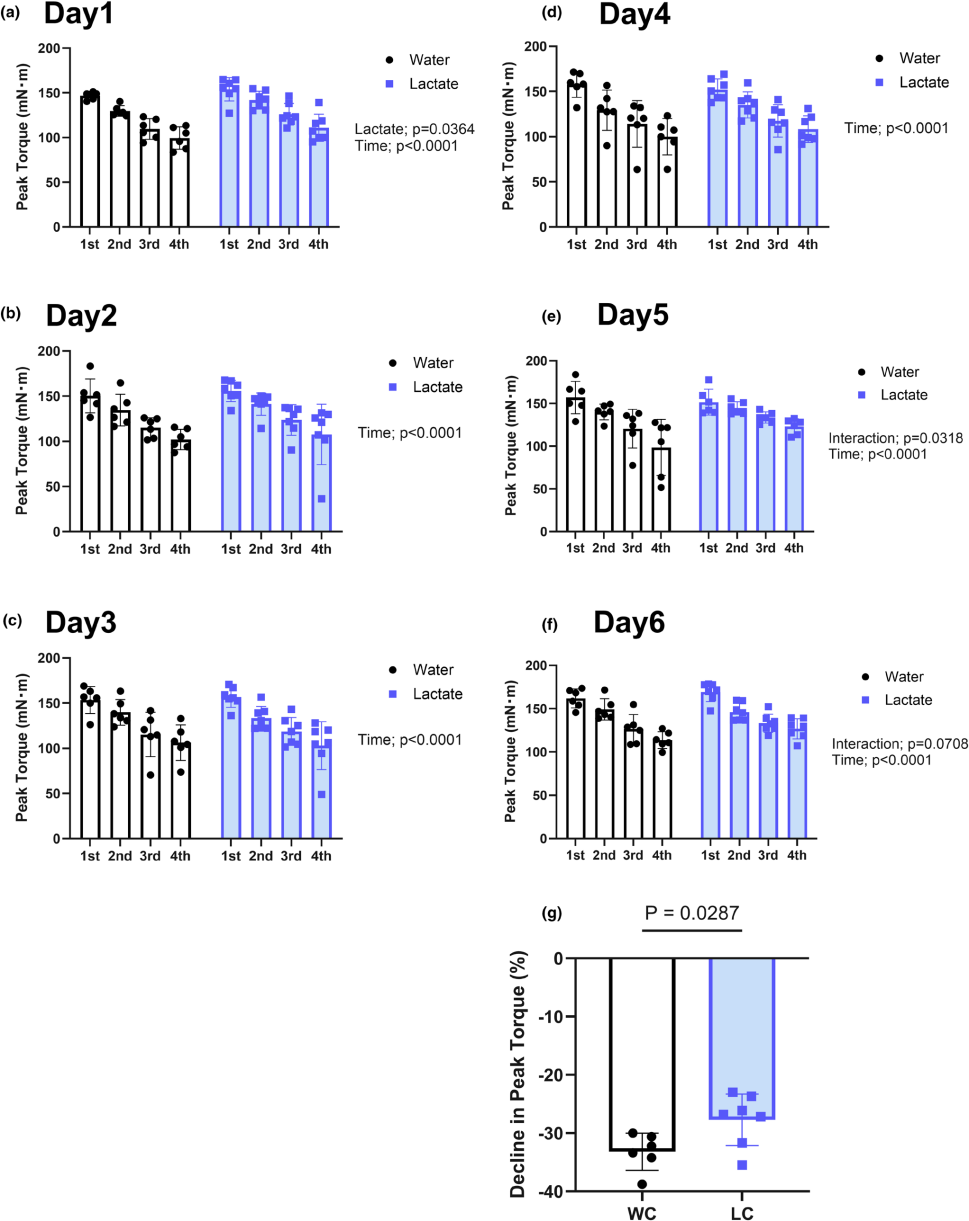
Peak torque and fatigue resistance during repeated stimulation. (a–f) Peak torque responses during the 1st to 4th sets on each training day (Days 1–6). (g) Average torque decrement ratio from set 1 to set 4 over six sessions as an index of fatigue resistance. Values are presented as the means ± standard deviation; *n* = 6 or 7 per group. The data were analyzed using a two‐way ANOVA and non‐paired *t*‐test.

Throughout the six chronic stimulation sessions, in all groups, we detected a progressive increase in peak torque values during each training day (main effect of time; Day 1–Day 6: all *p* < 0.05) (Figure 3a–f), indicating a training‐induced adaptation. On Day 1, lactate intake was found to promote a significant enhancement of peak torque production (main effect of lactate; *p* = 0.0364), whereas on Day 6, there was a trend toward a lactate × time interaction (*p* = 0.0708), thereby indicating a potential synergistic effect of lactate supplementation with repeated stimulation.

To assess fatigue resistance, we calculated the torque decrement ratio between the 1st and 4th sets within each session and averaged these among the six training sessions. This revealed that compared with rats in the control group, those in the lactate + chronic stimulation group (LC) were characterized by a significantly smaller reduction in torque (*p* < 0.05) (Figure 3g), thus indicating that an intake of lactate may contribute to attenuating fatigue during repeated high‐frequency contractions.

### 3.4 Muscle endurance performance and lactate threshold test

To determine whether a single oral dose of lactate affects resistance to local muscle fatigue or systemic endurance capacity, we conducted muscle endurance and lactate threshold (LT) tests under isometric conditions.

**FIGURE 4:**
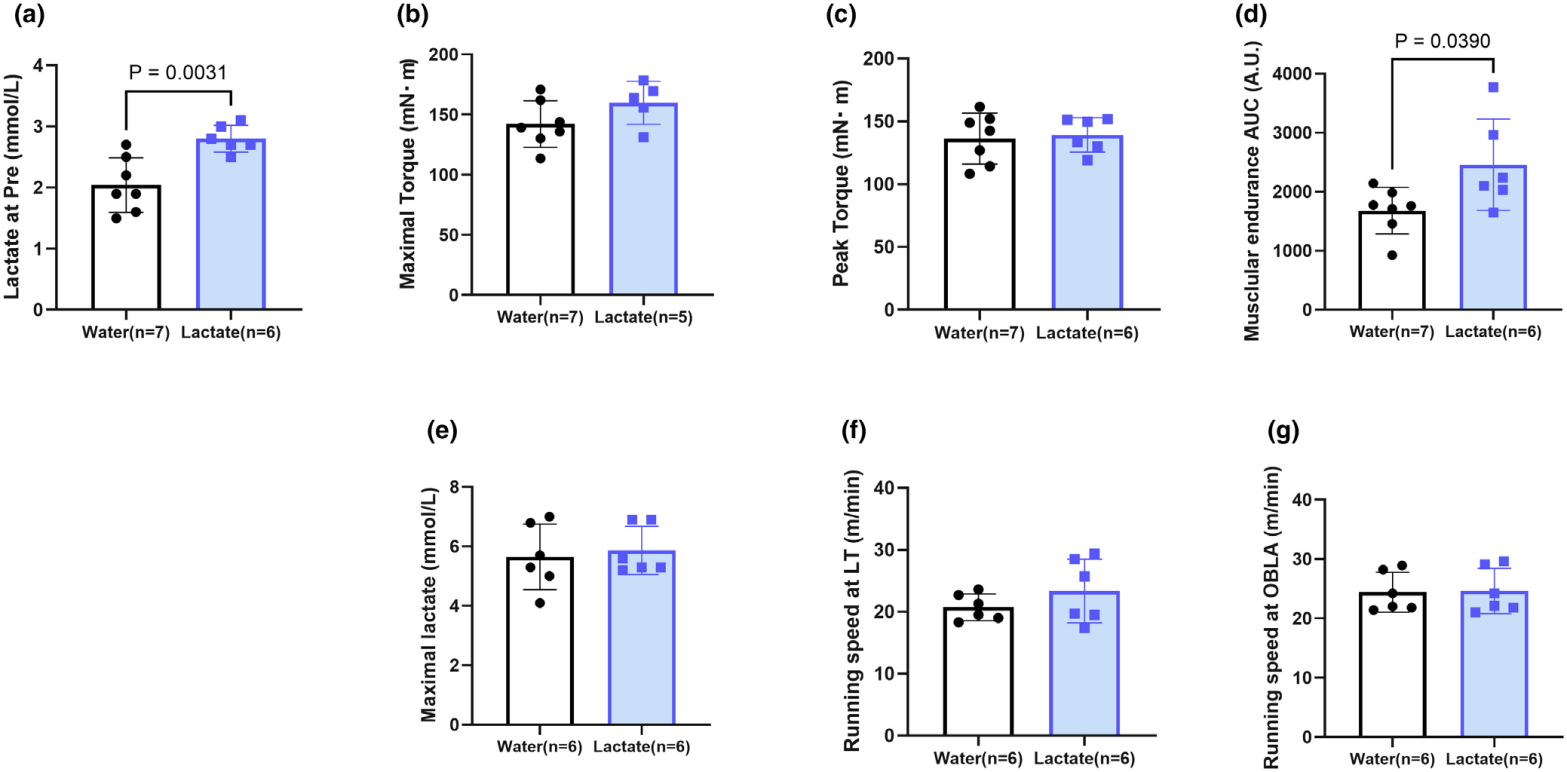
Effects of acute lactate intake on local muscle endurance and systemic endurance capacity. (a) Pre‐task blood lactate concentrations. (b) Maximal torque before the muscle endurance test. (c) Peak torque during stimulation. (d) Total work during 25 electrically induced contractions, calculated as the area under the torque‐time curve. (e) Maximum blood lactate concentrations during a treadmill lactate threshold (LT) test. (f) Running speed corresponding to the LT. (g) Running speed corresponding to the onset of blood lactate accumulation (OBLA). Values are presented as the means ± standard deviation; *n* = 5–7 per group. The data were analyzed using a non‐paired *t*‐test.

The findings of the muscle endurance test revealed a distinct local effect of lactate. Prior to the endurance task, lactate + chronic stimulation rats were found to have significantly elevated plasma lactate concentrations compared with those in the water group (*p* < 0.05) (Figure 4a), indicating the successful delivery of lactate. Although we detected no significant differences between the groups with respect to the maximal and peak torques during the fatigue task (Figure 4b,c), compared with rats in the water group, the total force production, quantified as the area under the torque‐time curve, was significantly higher in the lactate group (*p* < 0.05) (Figure 4d). These findings indicated that acute lactate intake contributed to enhancing fatigue resistance during repetitive contractions in skeletal muscle, independent of the peak force‐generating capacity.

Contrastingly, in the LT test, we detected no significant differences among groups regarding maximal blood lactate concentrations (Figure 4e), running speed corresponding to the LT (Figure 4f), or the OBLA (Figure 4g), thus indicating that chronic lactate supplementation did not alter the systemic endurance capacity, as assessed by a graded treadmill exercise.

### 3.5 Acute mTOR pathway activation is enhanced by lactate intake in oxidative muscle

**FIGURE 5:**
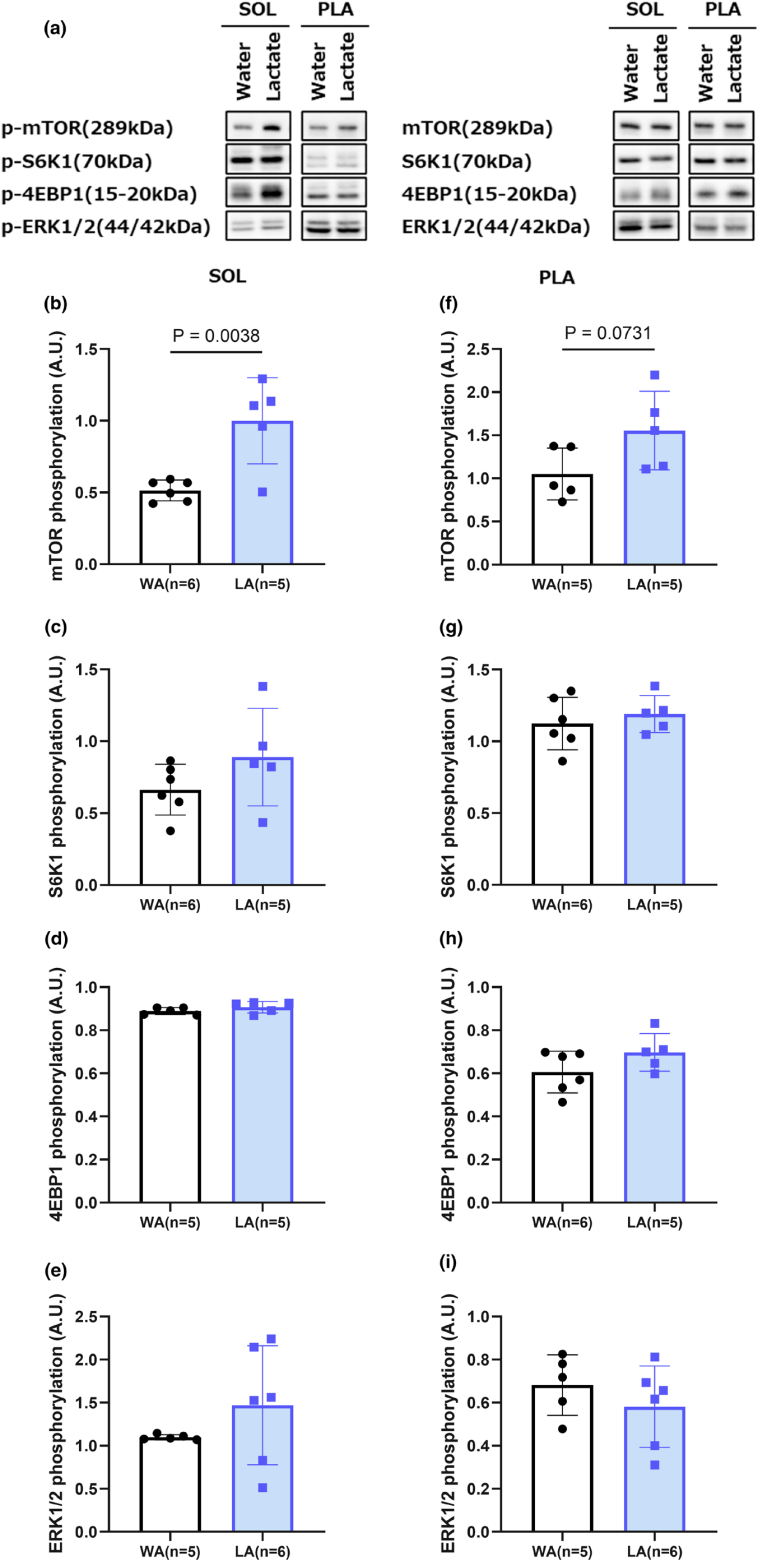
Effects of acute lactate intake on mTOR signaling in oxidative and glycolytic muscles. (a) Representative blots of phosphorylated and total proteins. (b–e) Quantification of the phosphorylated to total ratios of mTOR, S6K1, 4EBP1, and ERK1/2 in the soleus muscle. (f–i) Quantification of the same phosphorylated proteins (mTOR, S6K1, 4EBP1, and ERK1/2) in the plantaris muscle. Values are presented as the means ± standard deviation; *n* = 5 or 6 per group. The data were analyzed using a non‐paired *t*‐test. Additional details of the loading scheme, gel sectioning, membrane assembly, and uncropped immunoblots are provided in Figures S2 and S3.

To assess early anabolic and metabolic signaling events, we analyzed the activation of mTOR and energy‐sensing pathways following a single bout of electrical stimulation (Figure 5a–i).

In the soleus muscle, phosphorylation of mTOR was found to be significantly higher in the lactate + acute stimulation group (LA) compared with the control group (WA) (*p* = 0.0038) (Figure 5b), indicating the acute activation of the mTOR pathway in response to the ingestion of lactate. Although levels of phosphorylation of the downstream targets S6K1, 4EBP1, and ERK1/2 tended to be elevated in the LA group rats, these changes did not reach a level of statistical significance.

In contrast, although we detected no significant alterations in mTOR signaling in the plantaris muscle, the level of mTOR phosphorylation tended to increase among rats in the LA group (*p* = 0.0731) (Figure 5f), thereby indicating the possibility of a preferential fiber‐type‐specific response in oxidative muscles.

These findings accordingly indicated that an intake of lactate preferentially enhances anabolic mTOR signaling without markedly activating catabolic or stress‐associated metabolic pathways, at least under acute conditions, and that oxidative muscles may be more responsive to lactate in terms of early anabolic signaling.

### 3.6 Protein synthesis is enhanced by lactate in the myofibrillar fraction

**FIGURE 6:**
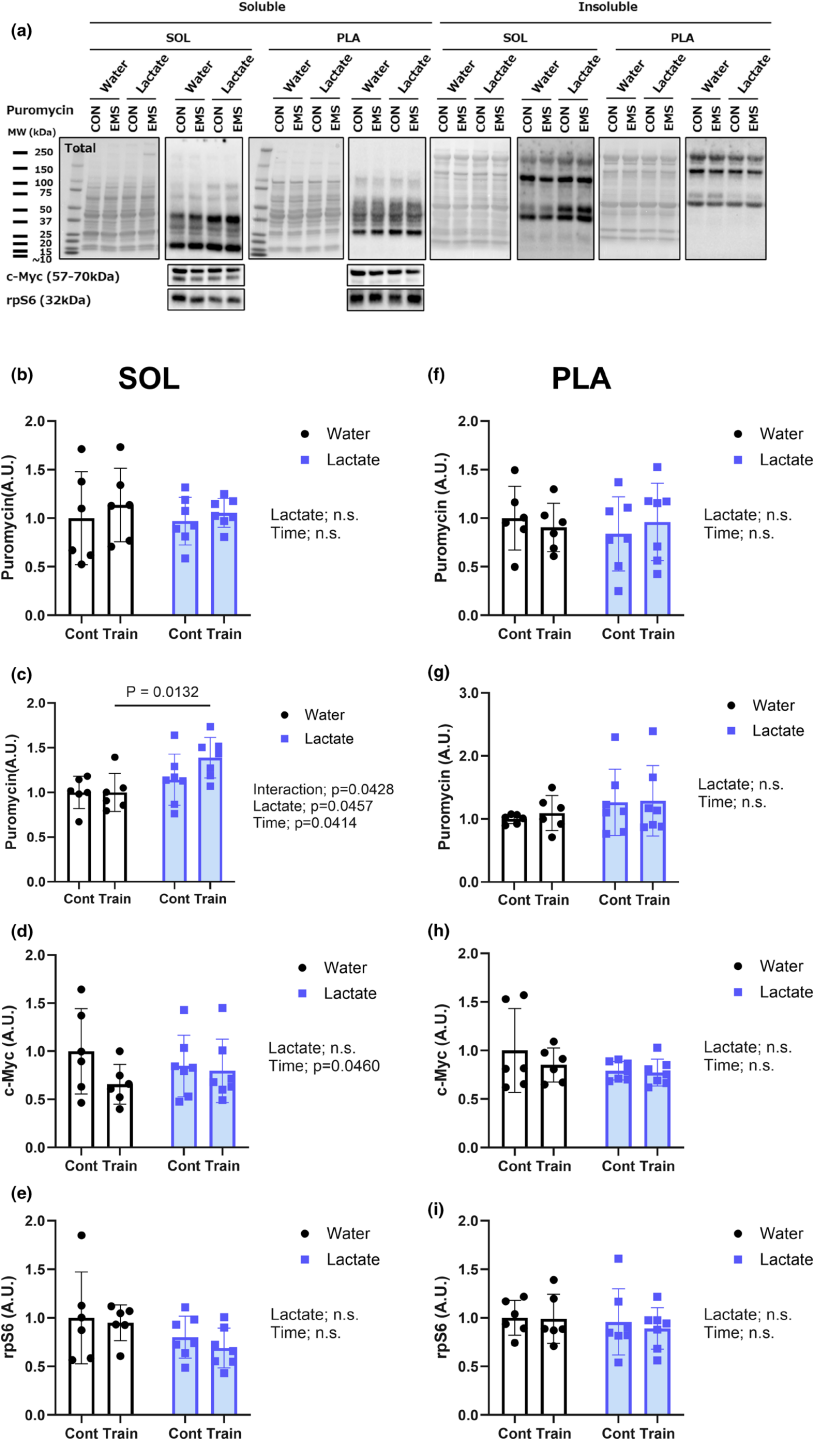
Lactate‐enhanced protein synthesis in the myofibrillar fraction of the soleus muscle. (a) Representative blots of puromycin‐labeled proteins in the soluble and insoluble fractions, and c‐Myc and rpS6 proteins in the soluble fraction. (b, c) Quantification of puromycin incorporation in the soluble (b) and insoluble (c) fractions of the soleus muscle. (d, e) Expression of translational regulators c‐Myc (d) and rpS6 (e) in the soluble fraction of the soleus muscle. (f, g) Quantification of puromycin incorporation in the soluble (f) and insoluble (g) fractions of the plantaris muscle. (h, i) Expression of translational regulators c‐Myc (h) and rpS6 (i) in the soluble fraction of the plantaris muscle. Puromycin incorporation was assessed in fractionated samples; therefore, labeling is not expected to appear uniformly across entire lanes, particularly in the myofibrillar fraction. Values are presented as the means ± standard deviation; *n* = 6 or 7 per group. The data were analyzed using a two‐way ANOVA followed by Tukey's post hoc test.

Compared to the rats in all other groups, we observed a significant increase in the incorporation of puromycin into the myofibrillar (insoluble) protein fraction of the soleus muscle in the LC group (*p* < 0.05), with a significant interaction (*p* = 0.0428) (Figure 6c). In contrast, no significant intergroup differences were observed with respect to the soluble fraction (Figure 6b) or the expression of c‐Myc or rpS6 (Figure 6d,e), although c‐Myc levels showed a significant main effect of time (*p* = 0.0460). These findings are consistent with the possibility that lactate intake is associated with greater myofibrillar puromycin incorporation into the soleus during chronic NMES, whereas c‐Myc and rpS6 levels did not show lactate‐related differences. In contrast, for the plantaris muscle, we detected no significant intergroup differences in puromycin incorporation in either protein fraction (Figure 6f–i), thereby indicating that the stimulatory effect of lactate on protein synthesis was more pronounced in oxidative muscle fibers. These findings should be interpreted as fraction‐specific incorporation rather than changes in whole‐muscle protein synthesis.

### 3.7 Lactate intake increases PCM1 protein abundance in the soleus muscle

**FIGURE 7:**
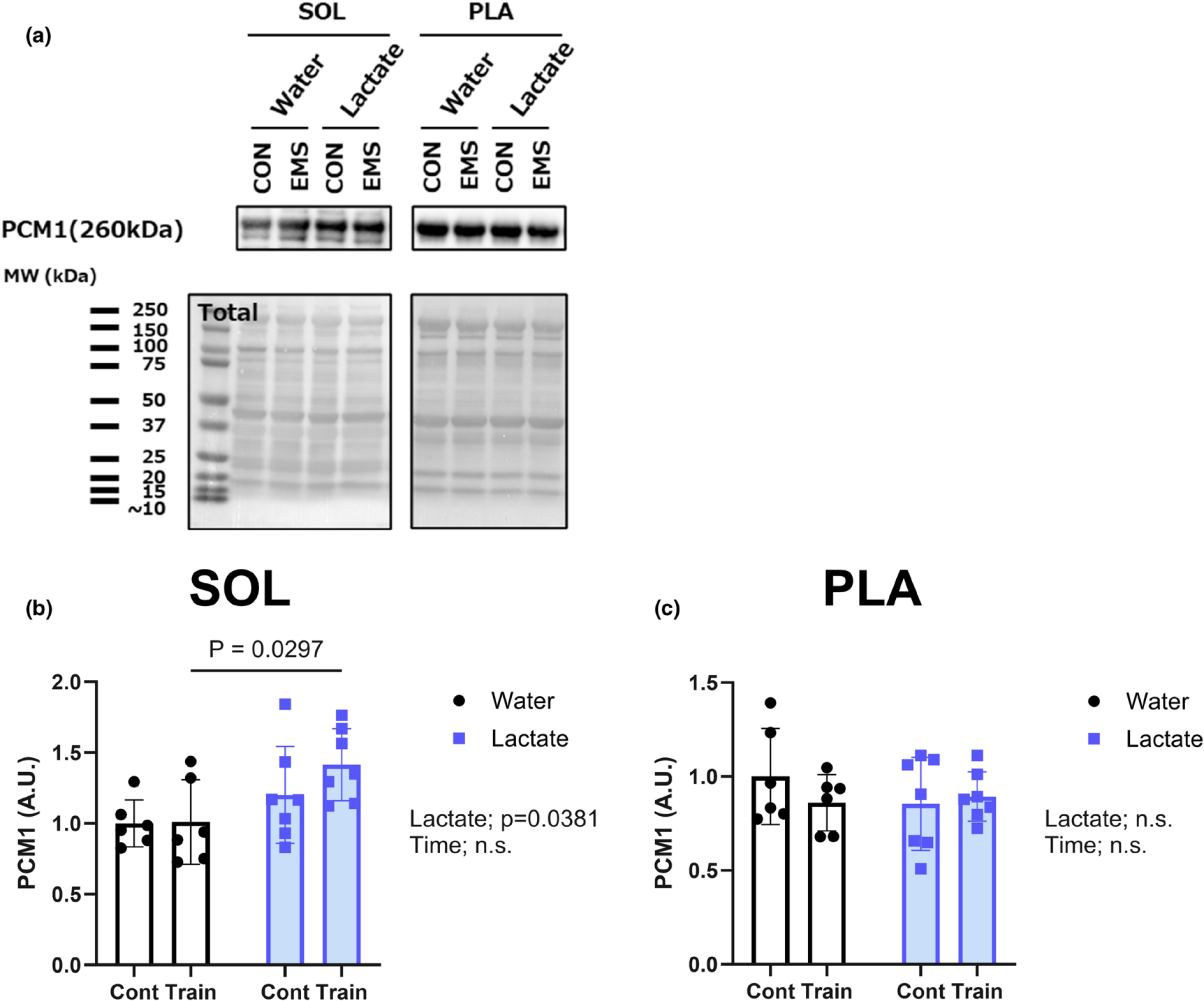
Expression of myonuclear markers in response to chronic lactate intake and electrical stimulation. (a) Representative blots of PCM1 in the soleus and plantaris muscles. (b, c) Values are presented as the means ± standard deviation; *n* = 6 or 7 per group. The data were analyzed using a two‐way ANOVA followed by Tukey's post hoc test.

An intake of lactate was found to be associated with a significant increase in PCM1 protein levels in the soleus muscle, as evidenced by a main effect of time (*p* = 0.0381), with post hoc analysis confirming higher PCM1 expression in the LC group rats compared with those in the WC group (*p* < 0.05) (Figure 7b). Comparatively, no notable changes in this regard were observed in the plantaris muscle, thus indicating fiber‐type‐specific changes in myonuclear‐related protein markers with lactate supplementation (Figure 7c).

### 3.8 Lactate enhances PGC‐1α expression in response to chronic training

**FIGURE 8:**
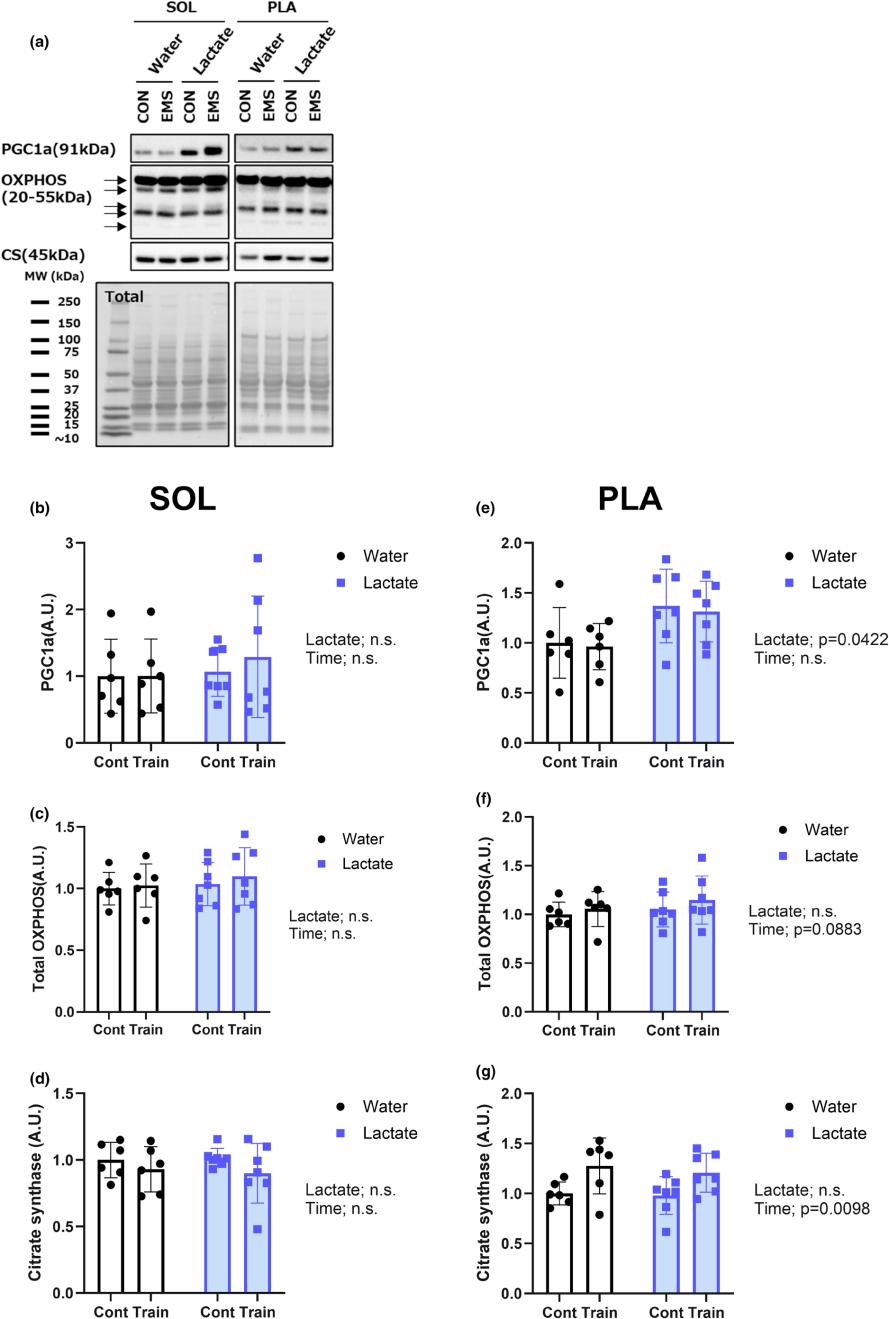
Lactate intake preferentially promotes the expression of mitochondrial biogenesis markers in glycolytic muscle. (a) Representative blots of PGC‐1α, OXPHOS complexes, and citrate synthase in the soleus and plantaris muscles. (b–d) Quantification of PGC‐1α (b), OXPHOS protein complex (c), and citrate synthase (d) in the soleus muscle. (e–g) Quantification of PGC‐1α (e), OXPHOS protein complex (f), and citrate synthase (g) in the plantaris muscle. Values are presented as the means ± standard deviation; *n* = 6 or 7 per group. The data were analyzed using a two‐way ANOVA.

In rats of the LC group, chronic stimulation combined with an intake of lactate elevated the expression of PGC‐1α (*p* = 0.0422) (Figure 8e) in the plantaris muscle. In addition, a significant effect of time was observed on citrate synthase levels (*p* = 0.0098; Figure 8g), and OXPHOS complex protein expression showed an increasing trend (*p* = 0.0883; Figure 8f). Taken together, these findings are consistent with the training‐induced mitochondrial adaptation in the plantaris muscle. In contrast, the soleus muscle showed no significant group differences regarding the expression of PGC‐1α, OXPHOS complexes, or citrate synthase (Figure 8b–8d). These findings indicate that lactate may preferentially promote mitochondrial‐related signaling in glycolytic muscles, even under resistance‐type electrical stimulation, whereas oxidative muscles, such as the soleus, may exhibit a ceiling effect or reduced sensitivity to such metabolic stimuli.

### 3.9 Expression of lactate transporters (MCT1/4) and receptor (GPR81)

**FIGURE 9:**
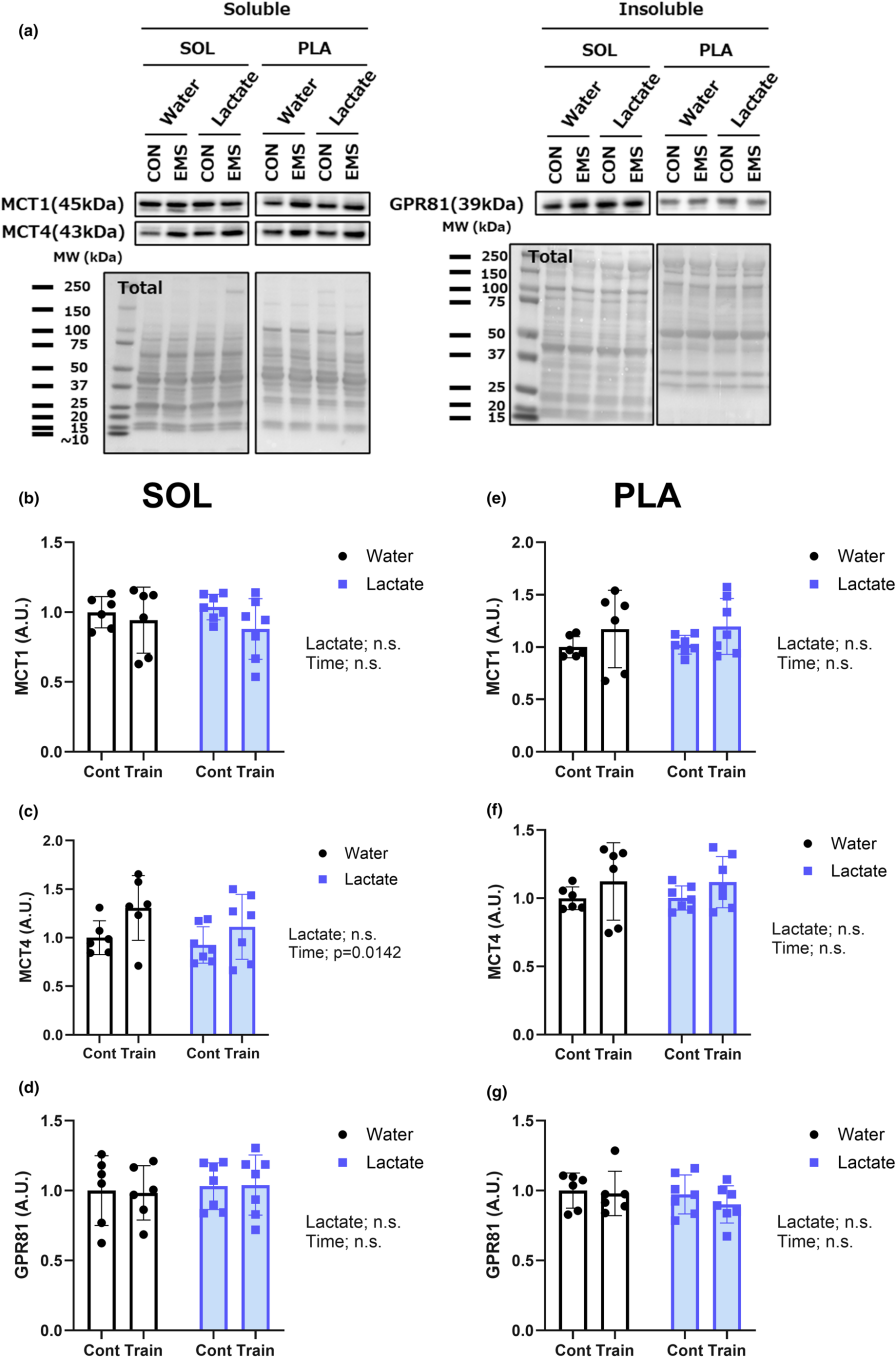
Time‐dependent changes in lactate transporter and receptor expression in response to chronic stimulation. (a) Representative blots of MCT1, MCT4, and GPR81 in the soleus and plantaris muscles. (b–d) Expression of MCT1 (b), MCT4 (c), and GPR81 (d) proteins in the soleus muscle. (e–g) Expression of MCT1 (e), MCT4 (f), and GPR81 (g) proteins in the plantaris muscle. Values are presented as the means ± standard deviation; *n* = 6 or 7 per group. The data were analyzed using a two‐way ANOVA.

With regards to the levels of MCT1 and GPR81 expression, we observed no significant changes in either the soleus (Figure 9b,d) or plantaris (Figure 9e,g) muscles following chronic stimulation either with or without an intake of lactate. In contrast, for the expression of MCT4, there was a significant main effect of time in the soleus muscle (*p* = 0.0142) (Figure 9c), indicating a training‐induced increase independent of lactate intake, whereas no comparable changes were detected in the plantaris muscle (Figure 9f). These findings thus indicate that the expression of lactate transporters and receptors is largely unaffected by lactate supplementation, with limited fiber‐type‐specific modulation of MCT4 expression being observed in oxidative muscle.

## 4 DISCUSSION

In this study, we determined whether oral lactate intake in male rats enhanced skeletal muscle adaptation to resistance‐type electrical stimulation. Although NMES alone elicited modest molecular responses, it induced significant functional adaptations, including increased torque and fatigue resistance, indicating that the stimulation paradigm was sufficient to elicit physiological adaptations. The main findings of this study are as follows: lactate intake (1) augmented muscle mass and torque output, particularly in the soleus muscle; (2) increased PCM1 abundance in oxidative muscle; (3) selectively promoted myofibrillar protein synthesis and acute mTOR signaling without influencing c‐Myc or rpS6; (4) induced mitochondrial‐related adaptations in glycolytic muscle; and (5) modulated MHC isoform expression patterns in a muscle‐type‐dependent manner. Collectively, these findings indicate that lactate acted as a metabolite‐derived signaling molecule associated with distinct molecular signatures and functional outcomes in skeletal muscles.

### 4.1 Lactate as an anabolic amplifier during resistance‐type stimulation

Traditionally regarded as a byproduct of anaerobic glycolysis, lactate has more recently emerged as a key signaling metabolite that regulates gene expression, mitochondrial biogenesis, and muscle regeneration (Brooks, 2020; Ohno et al., 2019). In the present study, we established that oral lactate supplementation augmented muscle mass and torque gain in response to resistance‐type electrical stimulation. These adaptations were accompanied by an increase in myonuclear‐associated PCM1 abundance and enhanced myofibrillar protein synthesis, thereby tending to indicate that lactate may influence the anabolic signaling milieu during electrically evoked contractions rather than a mere energy‐yielding substrate (Ohno et al., 2018; Takahashi et al., 2019). These findings support the concept of lactate as a myo‐metabolite that bridges mechanical loading and anabolic remodeling via specific signaling pathways, notably mTORC1 (Shirai et al., 2022).

Interestingly, the temporal pattern of lactate administration may play a key role in sustaining these adaptive responses. Unlike previous overload‐based studies in mice, in which lactate was administered daily throughout a chronic period (Shirai et al., 2022), in the model developed in the present study, lactate was administered only on training days (i.e., alternate days). Nevertheless, despite this intermittent schedule, the systemic lactate response, as evidenced by several markers showed increases or trends over the intervention period over a 6‐day period of monitoring, thereby indicating that when synchronized with mechanical activation, periodic exposure to lactate may prevent desensitization or metabolic saturation and thus facilitate prolonged molecular signaling. Notably, this intermittent design may reflect real‐world application scenarios more closely, and accordingly represents both an effective and safe approach for potential translational use. Although these adaptations were primarily observed in the soleus muscle, it is noteworthy that we also detected a nonsignificant increase in the mass of the plantaris muscle of rats in the lactate group (*p* = 0.0564). Despite the lack of statistical significance, this trend provides evidence that lactate may contribute to structural remodeling, even in the synergistic or secondary muscles involved in NMES. Given the enhanced torque observed, it is plausible that a minor gain effect on the plantaris muscle also contributed to enhancing force output, although at the present stage, this remains speculative in the absence of relevant cross‐sectional or electromyography data.

### 4.2 Fiber‐type‐specific regulation of myonuclear‐associated protein expression

PCM1 abundance was higher in the soleus muscle of the lactate‐supplemented group. Because PCM1 was quantified by immunoblotting in whole‐muscle homogenates, these measures cannot be interpreted as direct evidence of myonuclear accretion. Rather, the present data showed that lactate supplementation was associated with changes in myonuclear‐related protein markers in oxidized muscles during chronic NMES. This interpretation is partially supported by previous findings that myonuclear addition can occur independently of satellite cell expansion under hypertrophic conditions (Egner et al., 2016). By demonstrating that lactate intake combined with mechanical stimulation was associated with increased PCM1 expression in oxidative muscles, the present findings extend these observations to an in vivo context. Notably, these effects were more pronounced in the oxidative soleus muscle than in the glycolytic plantaris muscle, suggesting a fiber‐type‐specific sensitivity to lactate‐mediated anabolic signaling. This differential response may be attributed to the higher mitochondrial content and redox sensitivity of oxidative fibers, which enhanced signaling via lactate receptors or intracellular cofactors (Hashimoto et al., 2007; Schiaffino & Reggiani, 2011). Future studies using immunohistochemistry and/or single‐fiber analyses to directly quantify myonuclear number, satellite cell content, and fiber cross‐sectional area are required to determine whether lactate supplementation influences myonuclear addition and muscle growth under these conditions.

### 4.3 Lactate‐induced enhancement of Myofibrillar protein synthesis

Puromycin incorporation revealed that in response to chronic stimulation, lactate supplementation promoted a significant enhancement of myofibrillar (insoluble) protein synthesis in the soleus muscle. This selective stimulation of structural protein synthesis, without corresponding changes in the specific translational regulators assessed (c‐Myc and rpS6), indicates that lactate specifically augments translational efficiency in contractile protein networks. Consistent with this assumption, we observed a significant elevation in acute mTOR phosphorylation following an intake of lactate, with non‐significant trends observed in downstream targets (S6K1 and 4EBP1). These findings support the notion that lactate activates upstream anabolic signaling via mTORC1 (Goodman et al., 2010), and that this response is particularly prominent in slow‐twitch muscles subject to mechanical loading, which is consistent with previous evidence indicating that lactate can promote myogenic differentiation and anabolic signaling in vitro (Brooks, 2020; Ohno et al., 2018).

### 4.4 Fiber‐type‐specific mitochondrial signaling responses

An intake of lactate was also found to be associated with an increase in expression of the PGC‐1α protein, a key regulator of mitochondrial biogenesis, in the plantaris muscle, which is consistent with previous findings indicating that repeated intramuscular lactate injections (five times per week for 5 weeks) elevated the expression of PGC‐1α in the gastrocnemius muscles of rats (Zhou et al., 2021). In line with this observation, lactate intake was associated with improved fatigue resistance during local isometric contractions, while no changes were detected in systemic endurance capacity assessed by lactate threshold testing. This dissociation suggests a predominantly local, muscle‐specific metabolic effect of lactate. In contrast, we detected no significant inter‐group differences with respect to the levels of OXPHOS complex or citrate synthase proteins. However, a significant main effect of time for citrate synthase provided evidence that training alone can contribute to modest mitochondrial adaptations, whereas the trend‐level elevation of PGC‐1α may reflect the effects of lactate in enhancing early transcriptional priming. Nevertheless, it remains to be determined as to whether this priming is mediated by a direct effect of lactate or represents an indirect consequence of mechanical stimulation‐induced redox or energy stress. Notably, we were unable to detect comparable mitochondrial responses in the soleus muscle, which may indicate a ceiling effect in oxidative fibers in which the content of mitochondria is already high (Hashimoto et al., 2007; Little et al., 2011). These findings indicate that during electrically evoked contractions, lactate may preferentially promote mitochondrial gene expression in glycolytic muscle. However, under the present experimental conditions, we were unable to establish a definitive synergistic effect of lactate in conjunction with NMES with respect to mitochondrial‐related adaptations, which may have been attributable to the short duration of the intervention period or a sub‐maximal intensity of stimulation.

### 4.5 Changes in MHC composition as a structural adaptation

A chronic intake of lactate under conditions of electrically evoked training was found to promote a significant increase in the abundance of MyHC IIa protein in the soleus muscle, indicating a subtle shift in the contractile phenotype, even in this predominantly oxidative tissue. Given the fiber‐type composition of the soleus, a shift of this nature is particularly intriguing, and may reflect the capacity of this muscle to fine‐tune its phenotype in response to metabolic stimuli such as exogenous lactate. It is presumed that such adaptations are mediated by transcriptional regulators that are sensitive to redox and energy status, including PGC‐1α and MEF2, which have previously been implicated in fiber‐type modulation (Lin et al., 2002). In this regard, our observation of an increase in the IIa isoform may represent a refinement of oxidative‐glycolytic capacity, consistent with previous findings indicating that metabolic perturbation (e.g., via lactate) can promote an upregulation of oxidative gene programs (Hashimoto et al., 2007).

Contrastingly, with respect to MyHC isoform composition, despite a significant main effect of treatment, we detected no significant post‐hoc group differences for the plantaris muscle, which is characterized by a predominantly fast‐twitch fiber profile. This may thus indicate a trend toward slower remodeling kinetics or a higher plasticity threshold in fast‐twitch fibers in response to moderate mechanical and metabolic stimuli, although further studies are needed to confirm this phenomenon among different types of muscle and loading conditions (Adams et al., 1993; Talbot & Maves, 2016). The findings of previous studies in this regard have, nevertheless, provided evidence that lactate‐mediated signaling may preferentially influence oxidative muscle phenotypes (Certo et al., 2022; Summermatter et al., 2013). However, direct evidence for differences in fiber‐type shifts in red and white muscle, similar to our findings that the soleus, although not the plantaris, responded to lactate with contractile remodeling, remains limited.

### 4.6 Role of lactate transporters and receptors

Our findings that the levels of MCT1, MCT4, and GPR81 expression were not significantly altered in response to lactate intake would tend to indicate that the observed functional effects are probably mediated via pre‐existing transporter and receptor systems, rather than through an upregulation of transcription. As opposed to a direct response to exogenous lactate, we speculate that the time‐dependent increase in MCT4 expression observed in the soleus muscle may reflect a compensatory adaptation to an elevated glycolytic flux during repeated stimulation.

The findings of previous studies in this context have implicated the lactate receptor GPR81 (also referred to as HCAR1) in the modulation of ERK and mTOR signaling pathways, particularly in adipose and other non‐muscle tissues (Mohammad Nezhady et al., 2025; Cai et al., 2008). Although the expression of GPR81 remained unchanged in our model, it is plausible that the membrane‐localized activation of this receptor underlies the observed anabolic responses, including enhanced myonuclear‐associated protein abundance and mitochondrial‐related adaptations. Further investigations into the post‐translational regulation of GPR81 and the downstream signaling dynamics in skeletal muscle are accordingly warranted.

### 4.7 Limitations and future directions

Despite the important findings of this study, some limitations warrant consideration. First, we did not perform morphological assessments of the fiber cross‐sectional area or immunohistochemical analyses, which would have strengthened the interpretation of the structural adaptations. Second, gastrocnemius outcomes were not included in the present molecular analyses despite likely activation under the surface stimulation setup; therefore, muscle‐specific contributors to plantar flexion torque could not be fully determined. In preliminary assessments, gastrocnemius wet muscle mass did not change significantly following chronic NMES, whereas the soleus and plantaris muscles showed measurable adaptations. Subsequent molecular analyses were focused on these two muscles to investigate the mechanisms underlying the observed training‐ and lactate‐associated differences. Another consideration is the relatively modest stimulation volume employed in the present NMES protocol (four sets per session). This stimulation volume was selected based on previously validated NMES protocols in rodents, and was intentionally kept submaximal to avoid ceiling effects. Importantly, the aim of the present study was not to maximize NMES‐induced hypertrophy per se but rather to determine whether oral lactate supplementation could exert additive or synergistic effects under physiologically relevant resistance‐type loading conditions. Strong stimulation may obscure these modulatory effects by inducing near‐maximal anabolic responses that are independent of lactate availability. Accordingly, the present torque measurements reflect the combined effects of neural activation and changes in muscle size, and the intrinsic contractile properties normalized to the muscle cross‐sectional area could not be determined. Third, we did not fully delineate the temporal kinetics of mTOR effectors or transcriptional regulators, and further time‐course studies would help clarify lactate‐related signaling dynamics. Fourth, because stimulation was consistently applied to the left hindlimb, potential laterality effects cannot be completely excluded; however, all animals were treated identically, and the contralateral limb served as an internal control. Moreover, this study included only male rats, and given established sex differences in lactate metabolism and training responses, future studies in female animals are warranted. In addition, the study design did not include a lactate‐only group without NMES; therefore, we cannot determine whether the observed changes reflect lactate effects per se or interactions with electrically evoked contractions. Finally, functional assays of GPR81 activity or downstream redox signals (e.g., NAD^+^/NADH ratio) would further clarify the mechanistic pathways involved.

Nonetheless, despite these limitations, our findings provide evidence that oral lactate intake is associated with, and may facilitate, fiber‐type‐dependent molecular and functional adaptations during resistance‐type electrical stimulation. Lactate was shown to be associated with increased abundance of myonuclear‐associated proteins in oxidative muscle and promote mitochondrial‐related adaptations in glycolytic muscle, adaptations that coincided with changes in mTOR signaling and PGC‐1α transcriptional pathways, with minimal requirement for the upregulation of transporters or receptors. These findings highlight the role played by lactate as a metabolite‐derived signaling molecule that bridges metabolic stress and anabolic remodeling. From a translational perspective, lactate supplementation may provide a low‐burden, metabolically active adjunct to neuromuscular electrical stimulation, with potential applications in aging, rehabilitation, or clinical settings in which conventional exercise is limited.

## AUTHOR CONTRIBUTIONS

TY and HN conceived and designed the research. TY, YK, and SO performed the experiments. TY and YK analyzed the data. TY interpreted the results of the experiments. TY prepared the figures. TY drafted the manuscript. TY, YK, SO, and HN edited and revised the manuscript. All authors read and approved the final manuscript.

## FUNDING INFORMATION

This work was supported by the Japan Society for the Promotion of Science KAKENHI [grant numbers 23K20366 to T. Yoshihara]; and the Institute of Health and Sports Science & Medicine at Juntendo University.

## CONFLICT OF INTEREST STATEMENT

The authors declare that they have no competing interests.

## ETHICS STATEMENT

All experimental procedures were approved by the Ethics Committee for Animal Experiments at the Sakura Campus, Juntendo University (approval numbers 2024–26 and 2025–28), and were conducted in accordance with the principles for the care and use of laboratory animals stipulated by the Physiological Society of Japan.

## Supporting information


Figure S1.

Figure S2.

Figure S3.

Figure S4.

## Data Availability

The data supporting the findings of this study are available within the article and Figures [Link], [Link]. No additional datasets were generated or analyzed during the current study.
