# Supplementary material for: Lactate supplementation modulates molecular and functional responses during chronic neuromuscular electrical stimulation in male rats
Source: Physiol Rep. 2026 Mar 4;14(5):e70790. doi: 10.14814/phy2.70790 (PMC12960018; doi:10.14814/phy2.70790)
Supplement: Supplementary file 1 — Figure S1. [file PHY2-14-e70790-s004.docx]

**Figure S1. Time course of blood lactate concentrations following the oral administration of lactate.** Blood lactate concentrations were measured at 0, 10, 20, 30, and 60 min following oral intake of L‑sodium lactate (2 g/kg body weight; n = 7). Significant increases in the levels of lactate were detected at 10 min post‑administration and had approached a plateau level by 20 min (p < 0.05 vs. 0 min). These data were used to determine the timing of administration in the main experiments, wherein lactate was administered 15–20 min prior to electrical stimulation to ensure peak availability of systemic lactate. Data are expressed as the mean ± standard deviation. One-way repeated measures ANOVA with Tukey’s multiple comparisons test.
