# Supplementary material for: Lactate supplementation modulates molecular and functional responses during chronic neuromuscular electrical stimulation in male rats
Source: Physiol Rep. 2026 Mar 4;14(5):e70790. doi: 10.14814/phy2.70790 (PMC12960018; doi:10.14814/phy2.70790)
Supplement: Supplementary file 2 — Figure S2. [file PHY2-14-e70790-s002.docx]

**
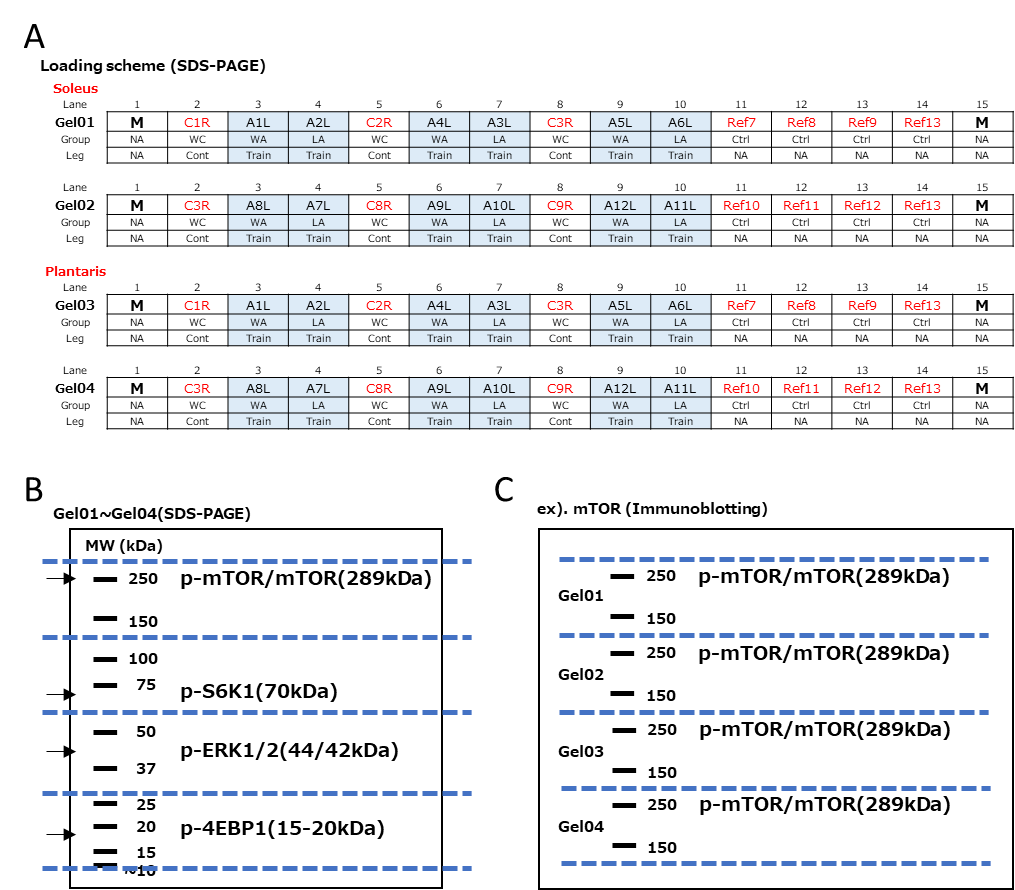
**

**Figure S2. Loading scheme, gel sectioning, and membrane assembly for immunoblot analyzes shown in Fig. 5A.** (A) A schematic representation of the sample loading scheme for phosphorylated and total mTOR, S6K1, ERK1/2, and 4EBP1 is demonstrated. (B) A single SDS–PAGE gel was sectioned into four regions according to approximate molecular weight ranges, and each region was used for immunoblotting with the corresponding antibodies. (C) The diagram also illustrates the cutting positions of the gel and the subsequent assembly of the gel fragments onto a single membrane for chemiluminescent detection.
