# Supplementary material for: Lactate supplementation modulates molecular and functional responses during chronic neuromuscular electrical stimulation in male rats
Source: Physiol Rep. 2026 Mar 4;14(5):e70790. doi: 10.14814/phy2.70790 (PMC12960018; doi:10.14814/phy2.70790)
Supplement: Supplementary file 3 — Figure S3. [file PHY2-14-e70790-s001.docx]

**
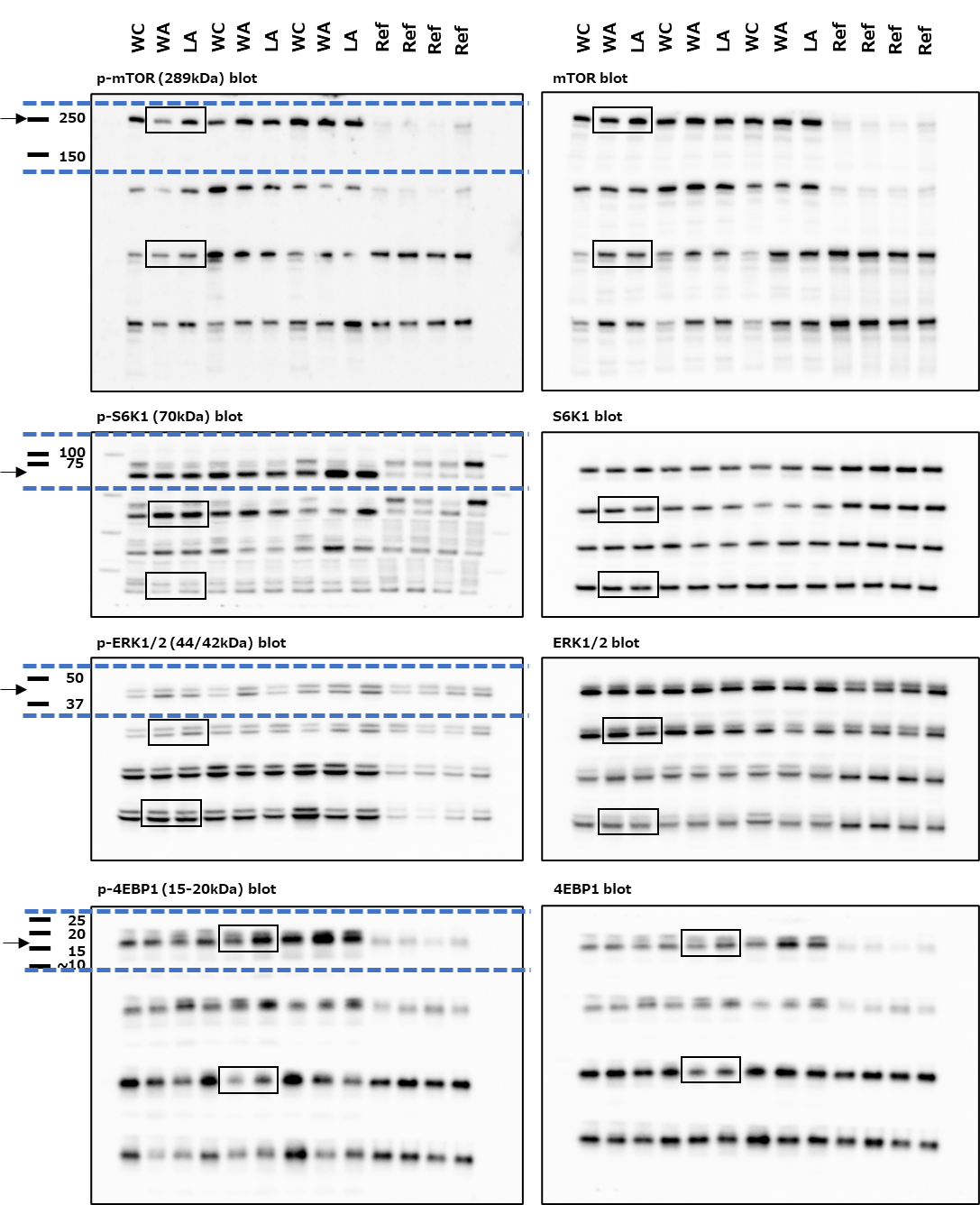
**

**Figure S3. Uncropped immunoblot images corresponding to Fig. 5A.** Representative full membrane images for phosphorylated and total mTOR, S6K1, ERK1/2, and 4EBP1 are presented. The regions utilised to generate the cropped panels presented in Fig. 5A are indicated by boxes. These images illustrate the arrangement of gel fragments on the membrane and provide transparency regarding the immunoblotting and image acquisition procedures.
