# Supplementary material for: Lactate supplementation modulates molecular and functional responses during chronic neuromuscular electrical stimulation in male rats
Source: Physiol Rep. 2026 Mar 4;14(5):e70790. doi: 10.14814/phy2.70790 (PMC12960018; doi:10.14814/phy2.70790)
Supplement: Supplementary file 4 — Figure S4. [file PHY2-14-e70790-s003.docx]

**
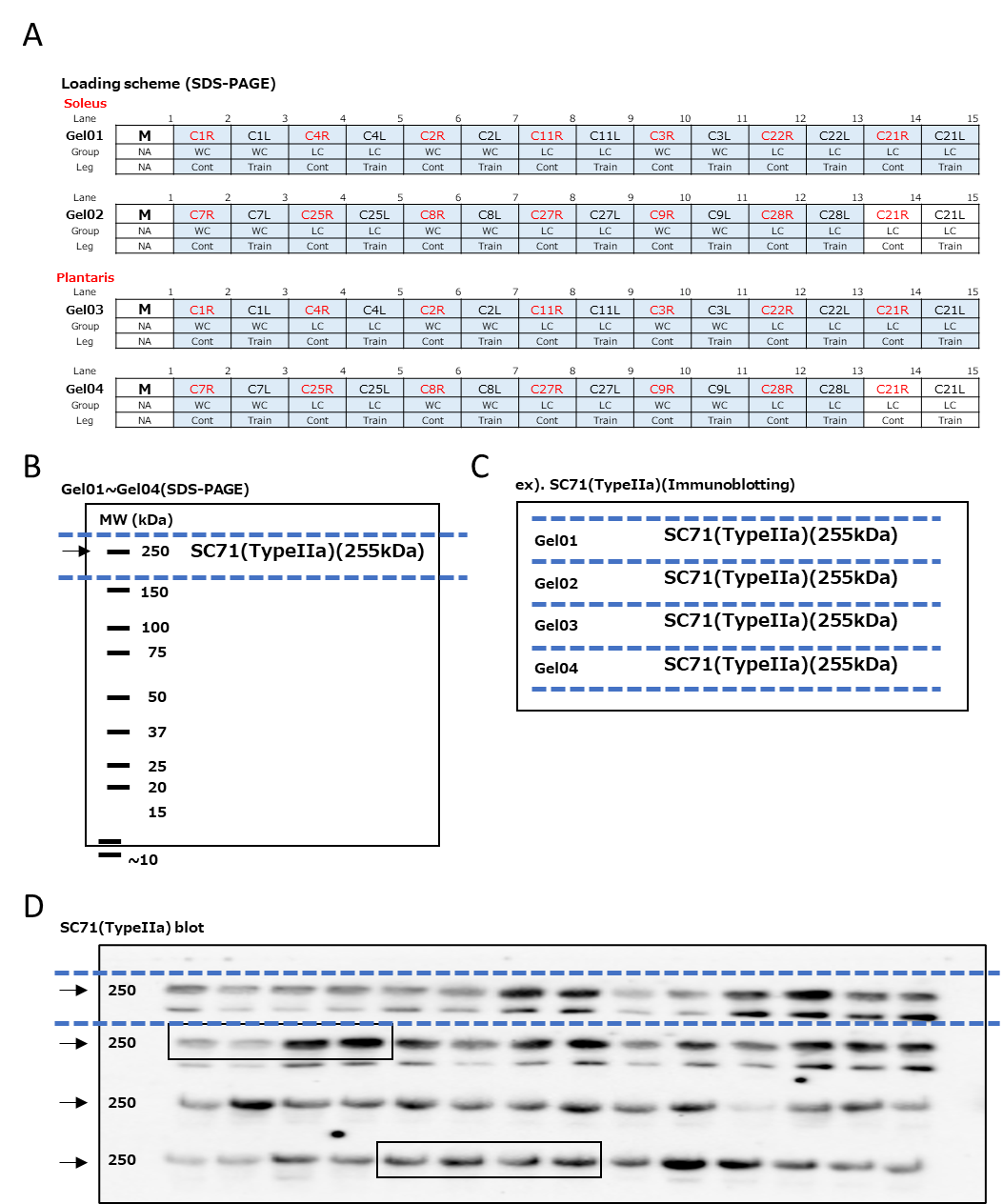
**

**Figure S4. Loading scheme, gel sectioning, membrane assembly, and uncropped immunoblot images corresponding to Fig. 1E.** (A) The following schematic illustration depicts the sample loading scheme for SC71 immunoblotting. (B, C) The illustration provides diagrams indicating the gel cutting positions and the arrangement of the gel fragments on the membrane. (D) Representative uncropped immunoblot images are also provided, with boxes indicating the regions used to generate the cropped panels presented in Fig. 1E. These data clarify the molecular weight regions analyzed and the membrane assembly strategy used for SC71 detection.
